# *Mesua beccariana* (Clusiaceae), A Source of Potential Anti-cancer Lead Compounds in Drug Discovery

**DOI:** 10.3390/molecules170910791

**Published:** 2012-09-10

**Authors:** Soek Sin Teh, Gwendoline Cheng Lian Ee, Siau Hui Mah, Yang Mooi Lim, Mawardi Rahmani

**Affiliations:** 1 Department of Chemistry, Faculty of Science, Universiti Putra Malaysia, 43400 UPM Serdang, Selangor, Malaysia; 2 Faculty of Medicine and Health Science, Universiti Tunku Abdul Rahman, 43000 Kajang, Selangor, Malaysia

**Keywords:** cyclodione, mesuadione, *in vitro*, cytotoxic, *Mesua beccariana*

## Abstract

An investigation on biologically active secondary metabolites from the stem bark of *Mesua beccariana* was carried out. A new cyclodione, mesuadione (**1**), along with several known constituents which are beccamarin (**2**), 2,5-dihydroxy-1,3,4-trimethoxy anthraquinone (**3**), 4-methoxy-1,3,5-trihydroxyanthraquinone (**4**), betulinic acid (**5**) and stigmasterol (**6**) were obtained from this ongoing research. Structures of these compounds were elucidated by extensive spectroscopic methods, including 1D and 2D-NMR, GC-MS, IR and UV techniques. Preliminary tests of the *in vitro* cytotoxic activities of all the isolated metabolites against a panel of human cancer cell lines Raji (lymphoma), SNU-1 (gastric carcinoma), K562 (erythroleukemia cells), LS-174T (colorectal adenocarcinoma), HeLa (cervical cells), SK-MEL-28 (malignant melanoma cells), NCI-H23 (lung adenocarcinoma), IMR-32 (neuroblastoma) and Hep-G2 (hepatocellular liver carcinoma) were carried out using an MTT assay. Mesuadione (**1**), beccamarin (**2**), betulinic acid (**5**) and stigmasterol (**6**) displayed strong inhibition of Raji cell proliferation, while the proliferation rate of SK-MEL-28 and HeLa were strongly inhibited by stigmasterol (**6**) and beccamarin (**2**), indicating these secondary metabolites could be anti-cancer lead compounds in drug discovery.

## 1. Introduction

**Figure 1:**
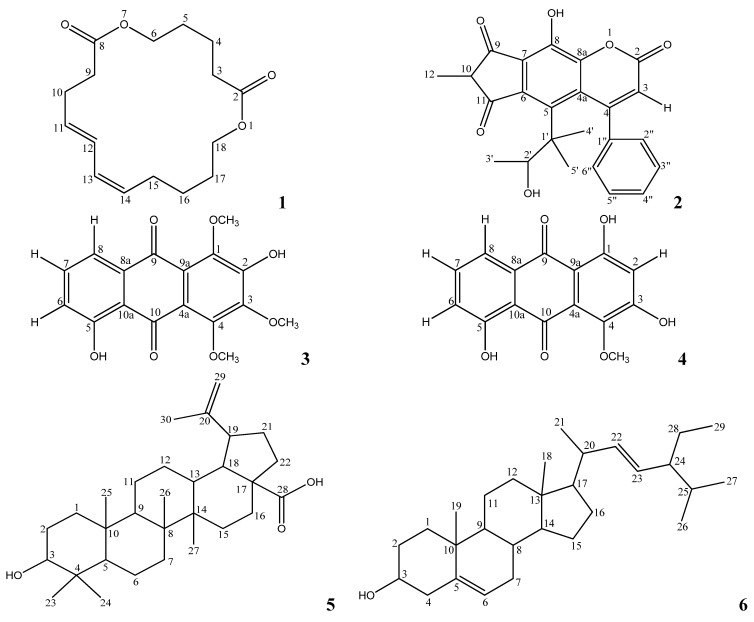
Structures of compounds.

Herbal plants are usually a primary source of medicines in many developing countries. One aspect of natural product research is the discovery of new drugs with resistance-reversing effects. *Mesua*, which is from the Clusiaceae family is a prolific producer of metabolites. Different parts of *Mesua* species are traditionally used as folk medicine for treatment of dyspepsia, fever, renal diseases and even as a poultice [1]. The flowers are traditionally used in conditions like asthma, cough and fever, whereas the fresh flowers are useful in reducing itchiness and nausea. It has been reported to exhibit various pharmacological activities such as antibiotic, neuromodulator, antitumor and antiviral activities [2]. Previous chemical investigations on this species have revealed their activities to be basically due to the presence in these plants of phloroglucinols, xanthones and neoflavonoids [3,4,5,6,7,8,9,10]. New potential anticancer agents which are important to the pharmaceutical industries have been identified from some *Mesua* species. These reports and the traditional uses of the *Mesua* species have led us to investigate the cytotoxic activities of *Mesua beccariana*. A new cyclodione, mesuadione (**1**), along with several known constituents **2**–**6** (Figure 1) were obtained from this ongoing research. The details of their cytotoxic activities are described in this manuscript.

## 2. Results and Discussion

Compound **1**, a new cyclodione, was successfully isolated from the hexane extract of the stem bark of *Mesua beccariana* as a colourless oil. The EIMS gave the molecular ion peak at *m*/*z* 280, indicating a molecular formula of C_16_H_24_O_4_. The UV and IR spectral data suggested the presence of unsaturation in the structure. The IR spectrum exhibited absorptions at 2927 and 2857 (CH_2_ and CH_3_ stretch), 1723 (C=O group), and 1177 (C–O stretch) cm^−1^ while the UV spectrum gave maximum absorptions at 217 and 230 nm. 

In the ^1^H-NMR spectrum (Table 1), signals attributed to twenty four hydrogens, which included *δ* 1.39 (*m*, 2H), 1.61 (*m*, 6H, overlapped, 2H × 3), 2.03 (*m*, 4H, overlapped, 2H × 2), 2.29 (*t*, 4H, overlapped, 2H × 2), 4.08 (*t*, 4H, overlapped, 2H × 2) and 5.29 (*m*, 4H, overlapped, 1H × 4). Moreover, sixteen carbons were seen in the ^13^C and DEPT experiments. These comprise two quaternary carbons (*δ* 174.1 × 2), four methines (*δ* 130.2 × 2 and 130.3 × 2) and ten methylenes (*δ* 25.4, 26.4, 27.2, 28.0, 29.2, 29.3, 35.0 × 2 and 64.3 × 2).

**Table 1:** ^1^H-NMR (400 MHz, CDCl_3_) and ^13^C-NMR (100 MHz, CDCl_3_) data for compound **1**.

Position	^1^H (*δ*)	^13^C (*δ*)	HMBC
1	−	−	−
2	−	174.1	−
3	2.29 (*t*, 2H)	35.0	C-2, C-4, C-5
4	1.61 (*m*, 2H)	25.4	C-2, C-3, C-5, C-6
5	1.61 (*m*, 2H)	29.2	C-3, C-6
6	4.08 (*t*, 2H)	64.3	C-5, C-8
7	−	−	−
8	−	174.1	−
9	2.29 (*t*, 2H)	35.0	C-8
10	2.03 (*m*, 2H)	28.0	C-11, C-12
11	5.29 (*m*, 1H)	130.3	C-9, C-10
12	5.29 (*m*, 1H)	130.2	C-10
13	5.29 (*m*, 1H)	130.2	−
14	5.29 (*m*, 1H)	130.3	C-16
15	2.03 (*m*, 2H)	26.4	C-13, C-14, C-17
16	1.39 (*m*, 2H)	27.2	C-14, C-17, C-18
17	1.61 (*m*, 2H)	29.3	C-15, C-18
18	4.08 (*t*, 2H)	64.3	C-2, C-17

**Figure 2:**
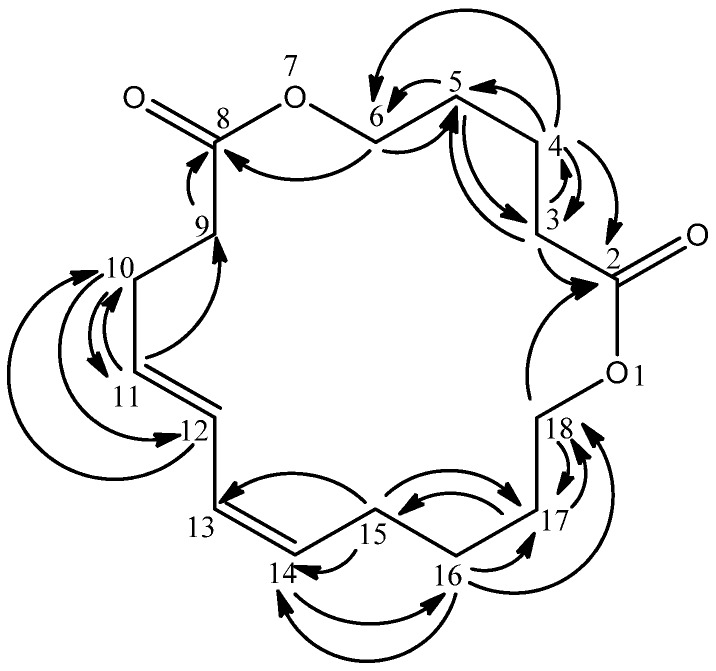
HMBC correlations in **1**.

The quaternary carbons were predicted to be carbonyl carbons due to their downfield chemical shifts as well as the appearance of C=O absorptions in the IR spectrum. Both carbonyl carbons were assigned as C-2 and C-8 due to long range HMBC correlations of C-3 and C-4 protons with the former carbonyl and C-6 proton with the latter carbonyl group (Figure 2). On the other hand, four vinylic protons *δ* 5.29 (H-11, H-12, H-13 and H-14) were observed in the ^1^H-NMR spectrum. H-11 revealed connectivities with *δ* 28.0 (C-10) and 35.0 (C-9); H-12 showed correlation with *δ* 28.0 (C-10) while H-14 correlated with *δ* 27.2 (C-16) as indicated in the HMBC experiment. Besides, the HMQC and HMBC spectra indicated that the overlapped proton at *δ* 2.03 (H-15) was directly bonded to *δ* 26.4 with long range correlations to *δ* 29.3 (C-17, ^3^*J*), 130.2 (C-13, ^3^*J*) and 130.3 (C-14, ^2^*J*). Hence, *δ* 2.03 was assigned as H-15; *δ* 2.03 was also directly bonded to *δ* 28.0 and correlated with *δ* 130.2 (C-12, ^3^*J*) and 130.3 (C-11, ^2^*J*) allowing its placement at C-10. The signal at *δ* 2.29 (H-9) was directly attached to *δ* 35.0 and displayed a ^2^*J* interaction with *δ* 174.1 (C-8). The connectivity to this downfield carbonyl signal confirmed its attachment to C-9.

The COSY spectrum revealed couplings of *δ* 1.61 to *δ* 1.39 (H-16) and 4.08 (H-18). The HMBC analysis showed correlations between *δ* 1.61 with *δ* 26.4 (C-15), and 64.3 (C-18) making it to be rightfully located at C-17. The H-17 signal was directly linked to *δ* 29.3 (C-17) from HMQC. The oxygenated methylene proton at *δ* 4.08 (H-18) displayed ^1^*J* coupling with *δ* 64.3 from HMQC and long range couplings to *δ* 29.3 (C-17) and 174.1 (C-2). This allowed its assignment to H-18.

Three overlapped protons (*δ* 1.61, 2.29 and 4.08) were alternatively assigned to three different positions due to their differences in their HMBC correlation values. The HMBC experiment indicated that *δ* 1.61 (H-5) correlated to *δ* 35.0 (C-3) and 64.3 (C-6); *δ* 1.61 (H-4) showed couplings to *δ* 29.2 (C-5), 35.0 (C-3), 64.3 (C-6) and 174.1 (C-2); *δ* 2.29 (H-3) correlates to *δ* 25.4 (C-4), 29.2 (C-5) and 174.1 (C-2) while *δ* 4.08 (H-6) correlated to *δ* 29.2 (C-5) and 174.1 (C-8). The structure of compound **1** was thus elucidated as 1,7-dioxacyclooctadeca-11,13-diene-2,8-dione, and it was given the common name mesuadione (Figure 1). Spectroscopic data for compounds **2**–**6** were in agreement with published data [9,11,12,13].

**Figure 3:**
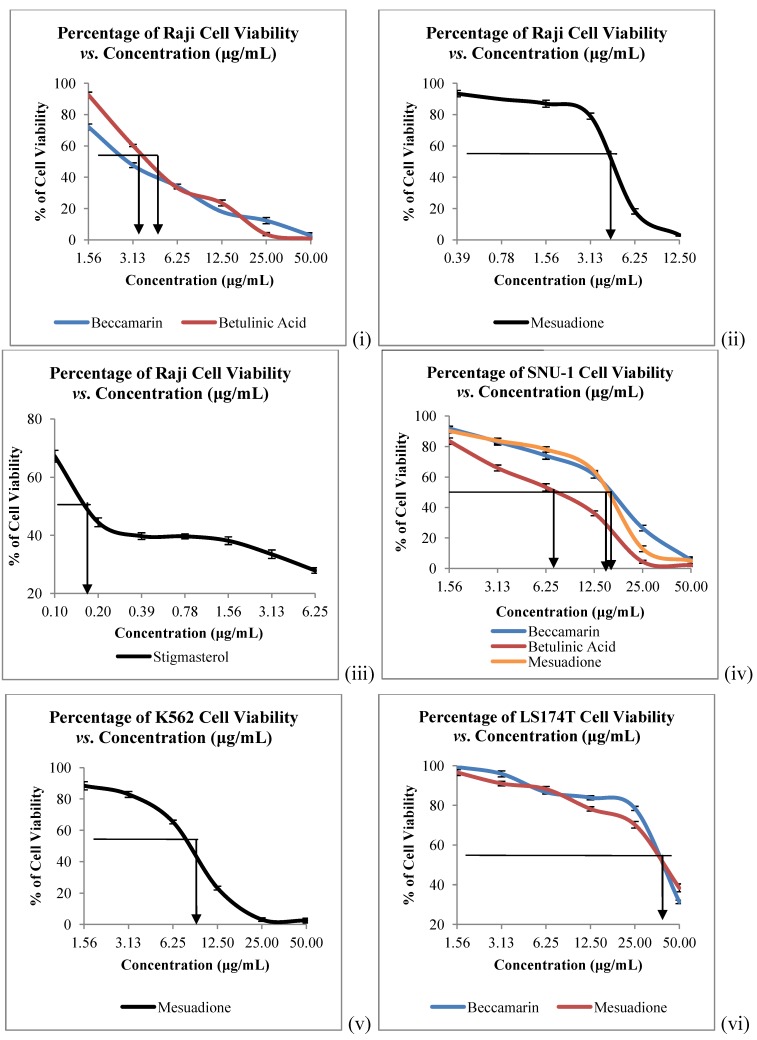
Cytotoxicity of Compounds **1**–**6** towards (i), (ii), (iii) Raji; (iv) SNU-1; (v) K562 and (vi) LS174T Cells. Bars denote statically significant differences at *p* < 0.05. Each data point represents the mean ± SD of three independent experiments.

**Figure 4:**
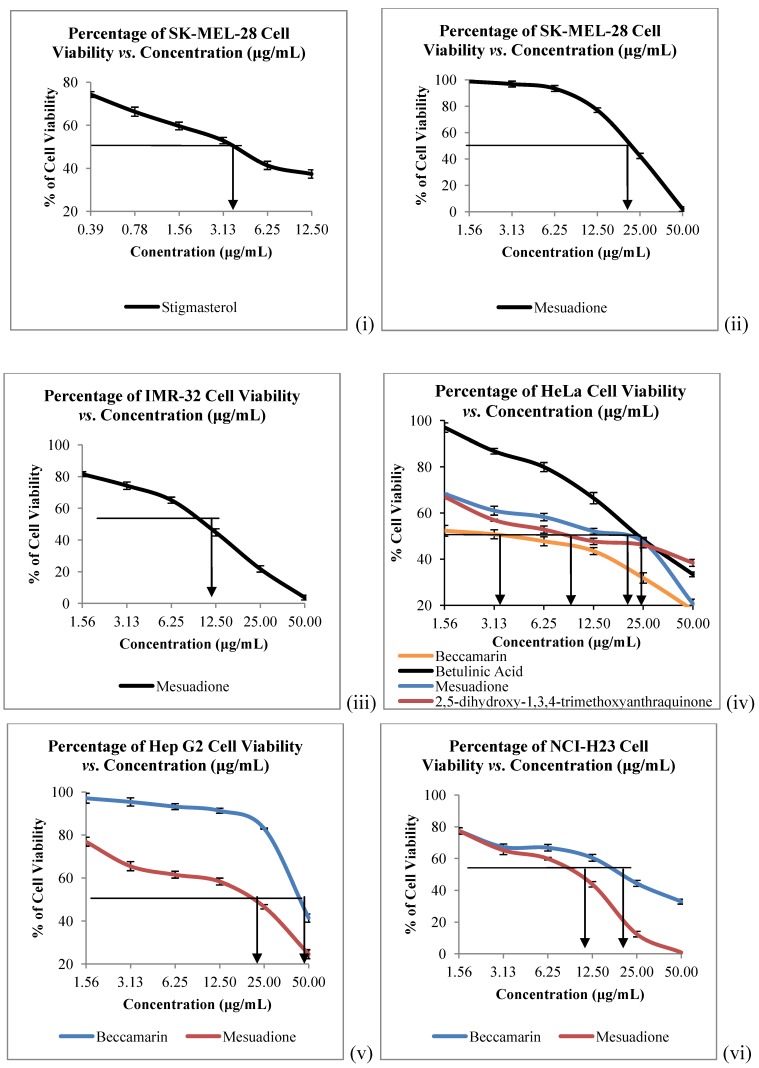
Cytotoxicity of Compounds **1**–**6** towards (i), (ii) SK-MEL-28, (iii) IMR-32; (iv) HeLa; (v) HEP G2 and (vi) NCI-H23 Cells. Bars denote statically significant differences at *p* < 0.05. Each data point represents the mean ± SD of three independent experiments.

The *in vitro* cytotoxicity of all the isolated constituents against nine cancer cell lines which are Raji (human B lymphocyte), SNU-1 (human gastric carcinoma), K562 (human erythroleukemia cells), LS-174T (human colorectal adenocarcinoma), HeLa (human cervical cells), SK-MEL-28 (human malignant melanoma cells), NCI-H23 (human lung adenocarcinoma), IMR-32 (human neuroblastoma) and Hep-G2 (human hepatocellular liver carcinoma) were evaluated. Compound **1** showed strong to weak cytotoxic activities against all the tested cancer cells, with IC_50_ values ranging from 4.58 to 40.00 µg/mL (Table 2, Figure 3 and Figure 4). Mesuadione (**1**), beccamarin (**2**), betulinic acid (**5**) and stigmasterol (**6**) displayed strong inhibition on the Raji cell proliferation with IC_50_ values less than 5 µg/mL. On the other hand, the proliferation of SK-MEL-28 and HeLa were strongly inhibited by pure stigmasterol (**6**) and beccamarin (**2**), respectively. Compound **3** moderately inhibited the growth of HeLa cells. Furthermore, compounds **2**, **3**, **5** and **6** displayed moderate to weak cytotoxic activity to the rest of the tested cell lines, as shown in Table 2. The cytotoxic activity tests for 4-methoxy-1,3,5-trihydroxyanthraquinone (**4**) indicated it to be inactive towards all the tested cancer cell lines. Kaempferol and quercetin were used as standard compounds throughout the experiments. 

**Table 2:** IC_50_ values of a Panel of Human Cancer Cell Lines Treated with Compounds **1**–**6** and standards.

Compounds	Cell lines with IC_50_ values (μg/mL)								
	Raji	SNU-1	K562	LS-174T	SK-MEL-28	IMR 32	HeLa	Hep G2	NCI-H23
Mesuadione (**1**)	4.58	15.62	8.60	40.00	21.87	10.62	20.83	21.80	10.42
Beccamarin (**2**)	2.92	16.56	−	40.00	−	−	3.91	43.75	20.31
2,5-Dihydroxy-1,3,4-trimethoxyanthraquinone (**3**)	−	−	−	−	−	−	9.30	−	−
Betulinic acid (**5**)	4.16	7.30	−	−	−	−	23.44	−	−
Stigmasterol (**6**)	0.17	−	−	−	3.90	−	−	−	−
Kaempferol	12.5	10.93	−	−	21.87	−	5.00	33.33	18.75
Quercetin	2.08	6.30	9.89	−	21.88	31.25	8.00	5.21	17.50

Note: IC_50_ more than 50 µg/mL indicates weak activity and is indicated by −. Each data point represents the mean of the three independent experiments (significant differences at *p* < 0.05).

## 3. Experimental

### 3.1. General

NMR spectra were obtained using a Unity JEOL 400 MHz FT-NMR spectrometer (Kuala Lumpur, Malaysia) using CDCl_3_ as solvent and tetramethylsilane (TMS) as internal standard. EIMS were recorded on a Shimadzu GCMS-QP5050A spectrometer (Kuala Lumpur, Malaysia). Infrared spectra were measured using the universal attenuated total reflection (UATR) technique on a Perkin-Elmer 100 Series FT-IR spectrometer (Kuala Lumpur, Malaysia). Ultraviolet spectra were recorded in EtOH on a Shimadzu UV-160A, UV-Visible Recording Spectrophotometer. 

### 3.2. Plant Material

The stem bark of *Mesua beccariana* was collected from the Sri Aman district in Sarawak, Malaysia. The plant material (herbarium specimen No. RG 211) was identified by Associate Professor Dr. Rusea Go, Biology Department, Faculty of Science, Universiti Putra Malaysia.

### 3.3. Extraction and Isolation

Air-dried and powdered *Mesua beccariana* stem bark (3 kg) was extracted successively with *n*-hexane (Hex), dichloromethane (DCM) and ethyl acetate (EA). The extracts were dried under reduced pressure using a rotary evaporator to obtain the corresponding hexane (15.6 g), dichloromethane (21.2 g) and ethyl acetate (15.8 g) extracts. Column chromatographic purification of the hexane extract gave a new constituent, mesuadione (**1**, 126 mg) and the coumarin beccamarin (**2**, 20 mg). Mesuadione (**1**) was obtained from repeated silica gel column chromatography and eluting with hexane–chloroform (4:1). Meanwhile, the dichloromethane extract afforded betulinic acid (**5**, 45 mg) and stigmasterol (**6**, 23 mg) while the ethyl acetate extract gave two anthraquinones identified as 2,5-dihydroxy-1,3,4-trimethoxyanthraquinone (**3**, 2 mg) and 4-methoxy-1,3,5-trihydroxyanthraquinone (**4**, 2 mg). 

### 3.4. Spectral Data

*Mesuadione* (**1**). Colorless oil. UV (EtOH) λ_max_ nm (log ε): 230 (4.25) and 218 (4.07). IR ν_max_ (cm^−1^): 2927, 2857, 1723 and 1177. MS *m/z* (rel. int.): 280 [M^+^] (12), 136 (12), 123 (20), 109 (33), 96 (82), 82 (100), 67 (80), 56 (10). For ^1^H- and ^13^C-NMR spectra, see Table 1.

*Beccamarin* (**2**). Yellow solid. m.p. 139.0–139.6 °C. UV (EtOH) λ_max_ nm (log ε): 209, 229, 281 and 348. IR ν_max_ (cm^−1^): 3400, 2971, 1741, 1605 and 1466. MS *m/z* (rel. int.): 406, 392, 377, 350, 349, 293, 43. The ^1^H and ^13^C-NMR (CDCl_3_) spectral data are consistent with published data [9].

*2,5-Dihydroxy-1,3,4-trimethoxyanthraquinone* (**3**). Orange solid. UV (MeOH) λ_max_ nm (log ε): 218, 276 and 410. IR ν_max_ (cm^−^^1^): 3400, 2920, 2840, 1660, 1630, 1540. MS *m/z* (rel. int.): 330, 315, 312, 297, 287, 272, 227, 58. The ^1^H and ^13^C-NMR (CDCl_3_) spectral data are consistent with published data [10].

*4-Methoxy-1,3,5-trihydroxyanthraquinone* (**4**). Orange solid. UV (MeOH) λ_max_ nm (log ε): 279, 320, 425, 470 and 485. IR ν_max_ (cm^−^^1^): 3420, 2920, 2860, 1720, 1630, 1470. MS *m/z* (rel. int.): 286, 268, 257, 243, 212, 180. The ^1^H and ^13^C-NMR (CDCl_3_) spectral data are consistent with the published data [11].

*Betulinic acid* (**5**). White solid. m.p. 290–291 °C. IR ν_max_ (cm^−1^): 2939, 1687, 1453, 1375. MS *m/z* (rel. int.): 456, 248, 207, 189, 121, 107, 95, 81, 69, 55. The ^1^H and ^13^C-NMR (CDCl_3_) spectral data are consistent with the published data [12].

*Stigmasterol* (**6**). White needles. m.p. 165–166 °C. IR ν_max_ (cm^−1^): 3399, 2939, 1457, 1374. MS *m/z* (rel. int.): 412, 255, 159, 145, 95, 81, 69, 55. The ^1^H and ^13^C-NMR (CDCl_3_) spectral data are consistent with the published data [13].

### 3.5. Culture of Cells

Nine cancer cell lines: Raji, SNU-1, LS-174T, HeLa, SK-MEL-28, NCI-H23, IMR-32, Hep-G2 and K562 were obtained from Universiti Tunku Abdul Rahman (UTAR). The Raji, SNU-1, K562, HeLa, Hep G2 and NCI-H23 cells were maintained in RPMI-1640 supplemented with 10% fetal bovine serum (FBS) while the LS-174T, SK-MEL-28 and IMR-32 were maintained in MEM supplemented with 10% FBS. All the cell lines were cultured in 75 cm^2^ T-flask and maintained at 37 °C in 5% CO_2_ humidified incubator. All cell lines were sub-cultured depending on the confluence of cells and observed under an inverted microscope for cell viability and checking for any contamination. Once the cells reach the confluent state, cell numbers were evaluated using the trypan blue staining method in a hemocytometer. All cell lines were deposited in American Type Culture Collection (ATCC). Besides, all the cell lines except for the Raji and HeLa were classified under the biosafety level 1 by ATCC.

### 3.6. MTT Assay

The cytotoxicity study was performed by the MTT assay as described by Mosmann [14]. Selected healthy cell lines with specified concentrations were harvested. Stock solutions were prepared for each crude extract and pure compounds by dissolving in DMSO or DMF and made up to a concentration of 20 mg/mL. Serial dilutions were carried out to give six different sub-stocks with concentrations of 200.00, 100.00, 50.00, 25.00, 12.50 and 6.25 µg/mL. 

An aliquot of 100 µL of each sub-stock with different concentrations were added to each well together with 100 µL of selected cells to give concentrations of 100.00, 50.00, 25.00, 12.50, 6.25 and 3.13 µg/mL and made up to a final volume of 200 µL in each well. Cells with no extracts (200 µL, untreated cell control—positive control) and 200 µL of medium only (blank medium—negative control) were prepared in the same plate. Samples and controls were prepared in triplicate.

The plate was then incubated for 72 h at 37 °C in 5% CO_2_ incubator. After 72 h, 20 µL of MTT solution was added to all the wells and incubated for 3 h in a 5% CO_2_ incubator. The plate was then spun at 3,000 rpm for 10 min. Supernatant from each well (160 µL) was discarded and then DMSO (160 µL) was added to dissolve the purple formazan crystals.

The absorbance of each well was determined using a microplate reader at 550 nm. The average absorbance of each crude extract was calculated and the average value was used to determine the percentage of cell viability by using the following formula:


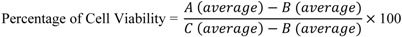


where: A = absorbance of sample

       B = absorbance of negative control

       C = absorbance of positive control

A graph of percentage of cell viability versus concentration was plotted for each extract and pure compound. The IC_50_ values were obtained from the plotted graph. Further dilutions were only performed on the compounds with IC_50_ values less than 3.13 µg/mL. Three independent experiments were conducted to assure the accuracy of the results. Kaempferol and quercetin were used as standard compounds throughout the cytotoxicity experiments.

## 4. Conclusions

A new cyclodione, mesuadione (**1**), was isolated from the stem bark of *Mesua beccariana* along with several known constituents **2**–**6**. Compounds **1**, **2**, **5**, and **6** were strongly cytotoxic towards the Raji cell line. These phytochemical and cytotoxicity investigations which demonstrated the cytotoxic effects of these compounds suggest them to be potential chemopreventive agents in drug discovery. 
